# Experimental and Theoretical Research on the Compression Performance of CFRP Sheet Confined GFRP Short Pipe

**DOI:** 10.1155/2014/109692

**Published:** 2014-02-03

**Authors:** Feng Li, Qilin Zhao, Li Chen, Guojian Shao

**Affiliations:** ^1^College of Mechanics and Materials, Hohai University, 1 Xikang Road, Nanjing 210098, China; ^2^PLA University of Science and Technology, 88 Houbiaoying Road, Nanjing 210007, China

## Abstract

The axial compressive strength of unidirectional FRP made by pultrusion is generally quite lower than its axial tensile strength. This fact decreases the advantages of FRP as main load bearing member in engineering structure. A theoretical iterative calculation approach was suggested to predict the ultimate axial compressive stress of the combined structure and analyze the influences of geometrical parameters on the ultimate axial compressive stress of the combined structure. In this paper, the experimental and theoretical research on the CFRP sheet confined GFRP short pole was extended to the CFRP sheet confined GFRP short pipe, namely, a hollow section pole. Experiment shows that the bearing capacity of the GFRP short pipe can also be heightened obviously by confining CFRP sheet. The theoretical iterative calculation approach in the previous paper is amended to predict the ultimate axial compressive stress of the CFRP sheet confined GFRP short pipe, of which the results agree with the experiment. Lastly the influences of geometrical parameters on the new combined structure are analyzed.

## 1. Introduction

Pultruded FRP (fiber reinforced composite) members are not limited on the length, can be made to be large-sectioned, and possess the advantages of lightness, corrosion resistance, and relatively low costs. As fibers in the pultruded FRP arrange unidirectionally, it has high axial tensile strength [1]. Therefore, pultruded FRP has been used as structural material for tensile members in many occasions instead of steel, such as the external tendon of prestressed concrete bridge [2] and the main cable of cable-stayed bridge [3]. On the other hand, pultruded FRP is also used for compression bearing members more and more widely, such as poles of light trusses [4], falsework [5], and other infrastructures [6]. Nevertheless, the compressive strength of pultruded FRP is quite lower than its tensile strength. So it may fail at much lower stress than its tensile strength. Hence, the ultimate bearing capacity under compression load condition is much smaller than that of strength condition, inducing great waste of material. As showed by the experiment on FRP poles carried by Zhang et al., the compressive strength of the CFRP (carbon fiber reinforced composite) poles and the GFRP (glass fiber reinforced composite) poles is only about 11% and 40% of their tensile strength, respectively, when there are completely no lateral confinement [7]. Zhou et al. research shows that the compressive strength of the GFRP poles is about 55% of their tensile strength [8]. Similar conclusions are given by Gao et al. [9], Yongzhi [10], and Wu [11]. As a result, the use efficiency of pultruded FRP as compressive members is quite low, and the popularization value is much poorer than that as tensile members. Yet there are abundant compressive members in actual engineering structures. Considering the numerous advantages and the abroad application prospect of pultruded FRP, people always wish to conquer, or partly conquer, its weakness by rational design and process and accordingly make efficient usage of it for engineering compressive members. Besides, sometimes the complexity of load condition in actual engineering also makes the compression of the primary tensile FRP members unavoidable. Take the bailey truss [12] for instance, when the truss is in work, its top chord bears compression and bottom chord bears tension, respectively. But because of the using demand that the two kinds of chords are able to be exchanged with each other, both kinds of chords should process quite high tensile strength and compressive strength synchronously. In other engineering structures, the stress states of the members may also change with the load condition and transfer the tensile members into compressive ones. Therefore, to heighten the compression strength of pultruded FRP by rational ways and accordingly broaden its application range is of great sense. Confining concrete members with CFRP sheet externally to enhance the compressive strength of the concrete is a widely adopted engineering technology currently [13, 14]. The mechanism is that the lateral surrounding confinement drives the axially compressed concrete into a state of multidirectional compression and subsequently restrains the lateral expansion and cracking of it. Analogously, experiments have showed that the axial compressive strength of unidirectional FRP can also be enhanced by lateral pressure applied on it [15]. For a kind of carbon/epoxy composite, its axial compressive strength increases by about 400 MPa when the hydrostatic pressure increases from 100 MPa to 500 MPa [16]. For a kind of glass/epoxy composite, its axial compressive strength increases by about 900 MPa when the hydrostatic pressure increases from 50 MPa to 300 MPa [17]. Based on this specialty of the unidirectional FRP and referring to the idea of CFRP sheet confined concrete, in the previous paper of the authors, the project to confine GFRP pole with surrounding CFRP sheet was proposed in order to heighten its axial compressive bearing capacity [18]. The project was experimentally verified and a theoretical iterative calculation approach was suggested to predict the ultimate compressive stress of the combined structure. Owning to its advantage on the global buckling stability, the GFRP pipe, namely, the GFRP hollow section pole, is more commonly used in actual engineering than the solid section GFRP pole. So experimental and theoretical research on the CFRP sheet confined GFRP short pipe was carried out in this paper on the basis of the previous paper.

## 2. Experiment

### 2.1. Experiment Process

**Figure 1:**
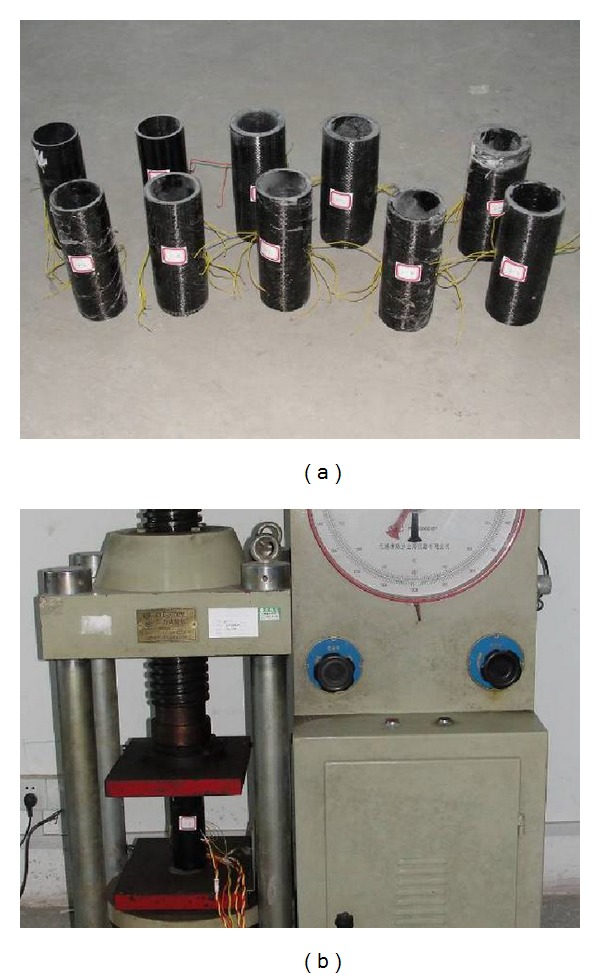
Test preparation of (a) specimens; (b) testing.

10 different CFRP sheet confined GFRP short pipe specimens were compressed (Figure 1). The GFRP pipes were produced by pultrusion, in which the fibers were all laid in the axial direction of the pipes. The CFRP sheet was produced by stitching tows of carbon fibers unidirectionally, of which the modulus was 200 GPa and the nominal thickness of the sheet layer was 0.167 mm. The measured initial mechanical parameters of the resin and the mechanical parameters of the glass fiber and the carbon fiber are showed in Table 1.

The inner radius and the length of the GFRP pipes were, respectively, 63 mm and 200 mm. There were two different wall thicknesses for the pipe, 4 mm and 8 mm. The carbon fiber sheet was cut along the laying direction of the fiber into strips, of which the width was equal to the length of the short pipes, wrapped on the short pipes after being dipped into the adhesive resin, and then solidified in room temperature. So the CFRP sheet confined GFRP pipes were finally attained.

The test was executed on a 200t compression testing machine. In the test, the data of compressive load, the axial strain, and the lateral strain was collected; the failure phenomenon was observed and recorded. The compressive load was directly read from the LCD of the testing machine. Each two strain gages which were laid transversely or axially were glued on the middle part of the specimen surface to get the strain values, and the values were then transmitted to a computer by a data acquisition system. The transverse and the axial strain gages were both laid symmetrically to the axis of the pole, in order to counteract the error induced by eccentricity. The prepared specimens and the testing situation are showed in Figure 1.

### 2.2. Experiment Result

**Figure 2:**
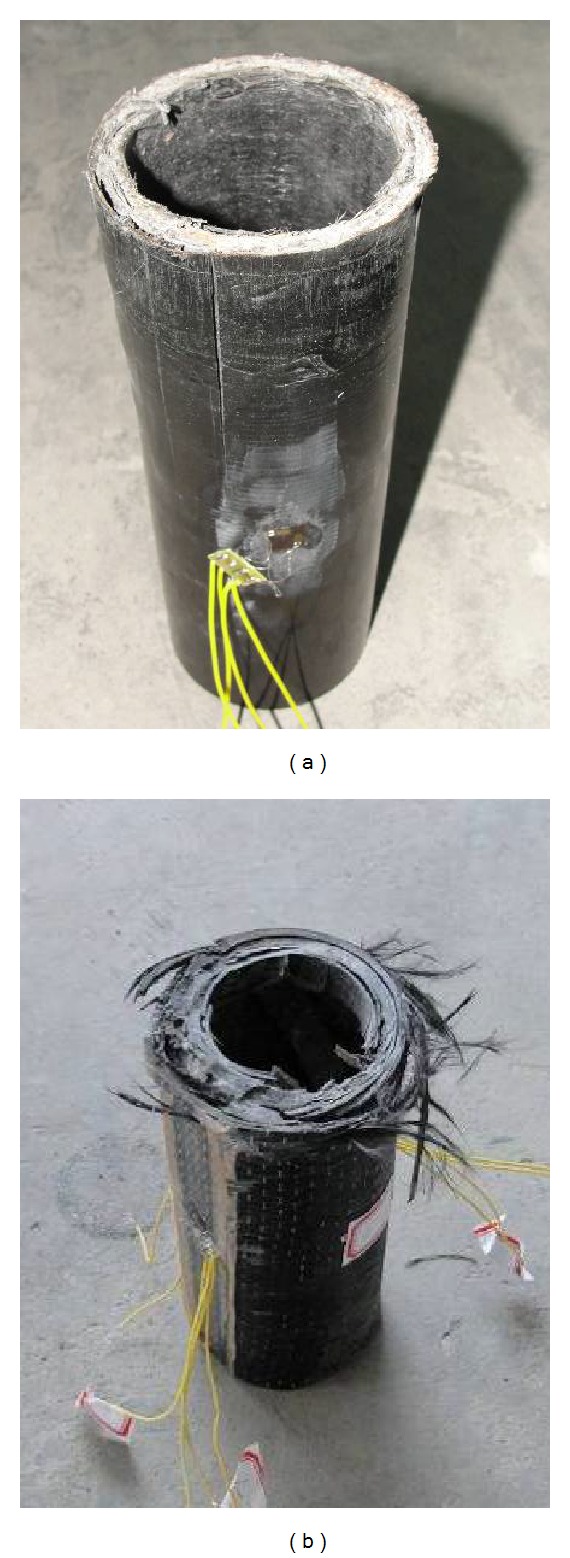
Compressing failed specimens of (a) compressing failed unconfined GFRP short pipe; (b) compressing failed CFRP sheet confined GFRP short pipe.

The unconfined GFRP short pipe gave a sudden crepitation as the load reached the ultimate value, and the load value decreased significantly at the same time, which was showed by the LCD. It could be seen that there were penetrating splits in the surface of the specimen. The specimen was then realized to be failing and the loading was terminated. The typical failure mode was longitudinal splitting of the GFRP, as showed by Figure 2(a).

The CFRP sheet confined GFRP short pipe gave a sudden crepitation as the load reached the ultimate value, and the load value decreased significantly at the same time. The CFRP sheet ruptured and the core GFRP pipe split more thoroughly than in the unconfined situation. The specimen was then realized to be failing and the loading was terminated. The typical failure mode was rupture of the CFRP sheet and subsequent longitudinal splitting of the GFRP, as showed by Figure 2(b).

The ultimate axial compressive stress of the specimens attained by experiment is listed in Table 2.

### 2.3. Preliminary Discussion

Comparing the results of different specimens in Table 2 it can be seen that, for the GFRP pipes of the same wall thickness, the ultimate axial compressive stress increases with the layers of the CFRP sheet. This indicates that the presence of the surrounding CFRP sheet can significantly increase the ultimate axial compressive stress of the GFRP pipe, which is similar with the situation of GFRP solid section pole confined by CFRP sheet.

From the failure phenomena described above it can be seen that the failure phenomena of the unconfined or CFRP confined GFRP short pipes are approximately consistent with the research on the GFRP solid section poles described in the previous paper [18]. So their mechanical behavior is the same as that of the solid section poles.

The macroscopic failure mode of the unconfined GFRP short pipe tends to be longitudinal splitting. When CFRP sheet confined GFRP pipe is axially compressed, it expands laterally because of Poisson ratio effect. This causes circumferential tensile strain and stress in the CFRP sheet, and then the CFRP sheet applies lateral pressure to the GFRP pipe conversely, which restrains the lateral expansion and longitudinal splitting of the short pipe and heightens its ultimate compressive stress.

When the axial compressive load applied on the CFRP sheet confined GFRP pipe increases, the circumferential tensile stress in the CFRP sheet will increase according to the lateral expansion of the GFRP pipe, and the confining stress to the GFRP pipe will increase thereupon, until the CFRP sheet ruptures. Although the combined structure is axially loaded, the core GFRP pipe is subjected to multiaxially compression, which affected by the axial load and the lateral confining pressure.

Before the CFRP sheet ruptures, the axial compressive load applied on the confined GFRP pipe has exceeded the ultimate load value in the unconfined situation, and the GFRP has reached the critical state of splitting. It does not fail just because the confinement from CFRP restrained the formation of macroscopic longitudinal splits in it. Once the CFRP sheet ruptured, the strain energy which is cumulated in the GFRP is released instantly and the pipe splits at the same time.

## 3. Theoretical Calculation Approach of Ultimate Compressive Stress

CFRP sheet is assumed to be a linear elastic material; its mechanical parameters can be considered to be insensitive to the stress state. In general condition, GFRP can be seen as a linear elastic material in the fiber direction, but under nonaxial stresses it can have a nonlinear performance [19–21]. That is because, in the two phases of materials that compose GFRP, the mechanical parameters of fiber cannot be affected by the stress state; yet those of the resin are sensitive to the stress state [15]. When the CFRP sheet confined GFRP pipe is axially loaded, the variation of stress state causes the variation of the resin mechanical parameters and accordingly the variation of the GFRP mechanical parameters.

In the previous paper, the theoretical iterative calculation approach considering the variation of GFRP mechanical parameters according to its stress state was proposed to predict the ultimate compressive stress of the CFRP sheet confined GFRP short pole. Based on this work, the theoretical iterative calculation approach for predicting the ultimate compressive stress of the CFRP sheet confined GFRP short pipe is established in this paper.

### 3.1. Elastic Mechanics Model

The equilibrium differential equations, the geometric equations, and the GFRP physical equations in cylindrical coordinate system are given as (1), (2) [22], and (3) [18], respectively:

(1) $$\begin{array}{c} \frac{\partial \sigma_{r}}{\partial r}+\frac{\partial \tau_{zr}}{\partial z}+\frac{\sigma_{r}-\sigma_{\theta }}{r}=0,\\ \frac{\partial \sigma_{z}}{\partial z}+\frac{\partial \tau_{rz}}{\partial r}+\frac{\tau_{rz}}{r}=0, \end{array}$$

(2) $$\begin{array}{c} \varepsilon_{r}=\frac{\partial u_{r}}{\partial r},\\ \varepsilon_{\theta }=\frac{u_{r}}{r},\\ \varepsilon_{z}=\frac{\partial w}{\partial z},\\ \gamma_{zr}=\frac{\partial u_{r}}{\partial z}+\frac{\partial w}{\partial r}, \end{array}$$

(3) [εzεrεθεrθεθzεzr] =[1E1−v12E2−v12E2000−v12E21E2−v23E2000−v12E2−v23E21E20000002(1E2+v23E2)0000001G120000001G12]  ×[σzσrσθσrθσθzσzr].

**Figure 3:**
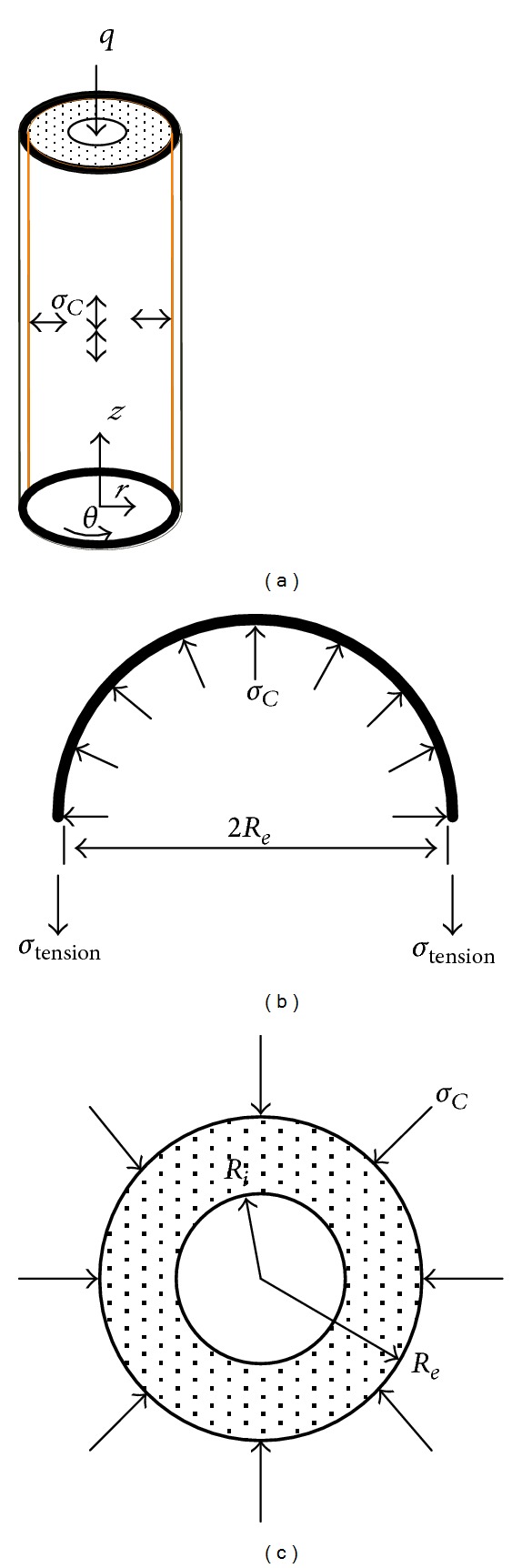
Mechanical model of the CFRP sheet confined GFRP short pipe: (a) combined structure; (b) forces equilibrium condition of the CFRP sheet; (c) forces equilibrium condition of the GFRP short pipe.

The mechanical model of the CFRP sheet confined GFRP short pipe is showed by Figure 3. Suppose that the core GFRP pipe is only affected by the lateral confining pressure *σ*_*C*_ without the axial compressive stress (Figure 3(c)); then the stress field boundary conditions are

(4) $$\begin{array}{c} \sigma_{z}=0,\\ \sigma_{zr}=\sigma_{rz}=0,\\ {\sigma_{r}|}_{r=R_{e}}=\sigma_{C},\\ {\sigma_{r}|}_{r=R_{i}}=0, \end{array}$$

where *R*_*e*_ and *R*_*i*_ are, respectively, the outer radius and the inner radius of the pipe section. Then from (3) we get

(5) $$\begin{array}{c} \varepsilon_{r}=\frac{\sigma_{r}-v_{23}\sigma_{\theta }}{E_{2}},\\ \varepsilon_{\theta }=\frac{\sigma_{\theta }-v_{23}\sigma_{r}}{E_{2}}. \end{array}$$

Exert the second formula of (4), while the first formula of (1) can be simplified as

(6) $$\begin{array}{c} \frac{\partial \sigma_{r}}{\partial r}+\frac{\sigma_{r}-\sigma_{\theta }}{r}=0. \end{array}$$

According to the first two formulas of (2), the deformation compatibility equation can be derived as

(7) $$\begin{array}{c} \frac{\partial \varepsilon_{\theta }}{\partial r}+\frac{\varepsilon_{\theta }{-\varepsilon }_{r}}{r}=0. \end{array}$$

Exert (3) to express the strains  *ε*_*θ*_ and *ε*_*r*_ in (7) with stress, and simplify it with (6), and the following equation can be attained:

(8) $$\begin{array}{c} \frac{\partial \left(\sigma_{r}+\sigma_{\theta }\right)}{\partial r}=0. \end{array}$$

Combine (6) and (8) and solve the simultaneous differential equations; then we get

(9) $$\begin{array}{c} \sigma_{r}=C_{1}+\frac{C_{2}}{r^{2}},\\ \sigma_{\theta }=C_{1}-\frac{C_{2}}{r^{2}}. \end{array}$$

By substituting (9) to the fourth formula of (4) we get

(10) $$\begin{array}{c} C_{1}+\frac{C_{2}}{R_{i}^{2}}=0⟹C_{1}=-\frac{C_{2}}{R_{i}^{2}}. \end{array}$$

By substituting the first formula of (9) and (10) to the third formula of (4) we get

(11) $$\begin{array}{c} C_{2}=-\frac{R_{e}^{2}R_{i}^{2}}{R_{e}^{2}-R_{i}^{2}}. \end{array}$$

Consequently we get the stress field in the GFRP pipe as

(12) $$\begin{array}{c} \sigma_{r}=\frac{\sigma_{C}R_{e}^{2}}{R_{e}^{2}-R_{i}^{2}}\left(1-\frac{R_{i}^{2}}{r^{2}}\right),\\ \sigma_{\theta }=\frac{\sigma_{C}R_{e}^{2}}{R_{e}^{2}-R_{i}^{2}}\left(1+\frac{R_{i}^{2}}{r^{2}}\right). \end{array}$$

When the axial compressive pressure *q* is applied to the combined structure of the CFRP sheet confined GFRP pipe, the radial deformation of the core GFRP pipe is composed of two parts, of which one is the deformation *u*_*rC*_^*F*^, caused only by the lateral confining pressure from the CFRP sheet, and the other is the deformation *u*_*rq*_^*F*^, caused only by the axial compressive load. The radial deformation *u*_*r*_^*C*^ of the CFRP sheet internal surface is equal to the radial deformation of GFRP pipe external surface, and this relation can be written as

(13) $$\begin{array}{c} u_{rC}^{F}+u_{rq}^{F}=u_{r}^{C}. \end{array}$$

Firstly only the affection of the lateral pressure *σ*_*C*_ on the GFRP pipe is considered. According to (3) and (12) we have

(14) $$\begin{array}{c} \varepsilon_{rC}^{F}=\frac{\sigma_{C}}{E_{2}}\frac{R_{e}^{2}}{R_{e}^{2}-R_{i}^{2}}\left(\left(1-\frac{R_{i}^{2}}{r^{2}}\right)-\left(1-v_{23}\right)\left(1+\frac{R_{i}^{2}}{r^{2}}\right)\right), \end{array}$$

where *ε*_*rC*_^*F*^ is the strain of GFRP pipe caused only by the lateral confining pressure. Consequently we get

(15) $$\begin{array}{c} u_{rC}^{F}=\overset{R_{e}}{\underset{R_{i}}{\int }}\varepsilon_{rC}^{F}\;dr=\frac{\sigma_{C}R_{e}\left(R_{e}-R_{e}v_{23}-R_{i}-R_{i}v_{23}\right)}{E_{2}\left(R_{e}+R_{i}\right)}. \end{array}$$

Then only the affection of axial pressure *q* on the GFRP pipe is considered, and we have

(16) $$\begin{array}{c} \varepsilon_{rq}^{F}=-\frac{qv_{12}}{E_{2}}, \end{array}$$

where *ε*_*rq*_^*F*^ is the radial strain of GFRP pipe caused only by the axial compressive load. Consequently we get

(17) $$\begin{array}{c} u_{rq}^{F}=\overset{R_{e}}{\underset{R_{i}}{\int }}\varepsilon_{rq}^{F}\;dr=-\frac{qv_{12}}{E_{2}}\left(R_{e}-R_{i}\right). \end{array}$$

By force equilibrium of the CFRP sheet we have

(18) $$\begin{array}{c} \sigma_{C}=-\frac{\sigma_{\text{tension}}t}{R_{e}}, \end{array}$$

where *σ*_tension_ is the tensile stress in the CFRP sheet and *t* is the total nominal thickness of the CFRP sheet layers. The radial deformation of CFRP can be written as

(19) $$\begin{array}{c} {u_{r}^{C}=R}_{e}\frac{\sigma_{\text{tension}}}{E_{\text{cf}}}+\frac{1}{2}tv_{\text{cf}}\frac{\sigma_{\text{tension}}}{E_{\text{cf}}}=-\frac{\sigma_{C}R_{e}^{2}}{tE_{\text{cf}}}\left(1+\frac{tv_{\text{cf}}}{2R_{e}}\right), \end{array}$$

where *E*_cf_  and *v*_cf_ are, respectively, the elastic modulus and the lateral Poisson ratio of the CFRP sheet. In general situation, because the value of *tv*_cf_/2*R* is much smaller than 1, it can be approximately considered that

(20) $$\begin{array}{c} u_{r}^{C}=-\frac{\sigma_{C}R_{e}^{2}}{tE_{\text{cf}}}. \end{array}$$

Substitute (16), (18), and (20) into (13) and we get

(21) $$\begin{array}{c} \sigma_{C}=\frac{v_{12}qE_{\text{cf}}t\left(R_{e}^{2}-R_{i}^{2}\right)}{R_{e}^{2}E_{2}\left(R_{e}+R_{i}\right)+R_{e}^{2}E_{\text{cf}}t\left(1-v_{23}\right)-R_{e}R_{i}E_{\text{cf}}t\left(1+v_{23}\right)}. \end{array}$$

According to (12) and (21) the internal stress field of the GFRP pipe can be summarized as follows:

(22) $$\begin{array}{c} \sigma_{r}=\frac{v_{12}qE_{\text{cf}}tR_{e}\left(1-R_{i}^{2}/r^{2}\right)}{R_{e}E_{2}\left(R_{e}+R_{i}\right)+E_{\text{cf}}tR_{e}\left(1-v_{23}\right)-E_{\text{cf}}tR_{i}\left(1+v_{23}\right)},\\ \sigma_{\theta }=\frac{v_{12}qE_{\text{cf}}tR_{e}\left(1+R_{i}^{2}/r^{2}\right)}{R_{e}E_{2}\left(R_{e}+R_{i}\right)+E_{\text{cf}}tR_{e}\left(1-v_{23}\right)-E_{\text{cf}}tR_{i}\left(1+v_{23}\right)},\\ \sigma_{z}=q. \end{array}$$

By substituting (22) into (3) the internal strain field of the pipe can be calculated as

(23) εθ=−qv12(E2r2Re(Re+Ri)−EcftReRi2  ×(1+v23)−EcftRir2(1+v23))  ×(E2r2(ReE2(Re+Ri)+EcftRe(1−v23)−EcftRi(1+v23)))−1,εr=−qv12(E2r2Re(Re+Ri)+EcftReRi2  ×(1+v23)−EcftRir2(1+v23))  ×(E2r2(ReE2(Re+Ri)+EcftRe(1−v23)−EcftRi(1+v23)))−1,εz=−q(−E22Re(Re+Ri)−E2EcftRe(1−v23)  +E2EcftRi(1+v23)+2v122ReEcftE1)  ×(E2E1(ReE2(Re+Ri)+EcftRe(1−v23)  −EcftRi(1+v23)))−1.

When *R*_*e*_ = *R* and *R*_*i*_ = 0, (22) and (23) will transform to the internal stress equation and the internal strain equation of the CFRP sheet confined GFRP solid section pole, respectively. This indicates that the CFRP sheet confined GFRP solid section short pole is special case of the CFRP sheet confined GFRP short pipe.

### 3.2. Nonlinear Property of GFRP

The elastic mechanics model of CFRP sheet confined GFRP pipe is established and the stress and strain distribution are calculated above. Yet the above model is based on the assumption that the GFRP material is always linear and elastic, without considering the nonlinear property under multiaxial compression of GFRP. Hence the calculated results by this model are not accurate enough. In order to attain more accurate results, the affection of multiaxial compression on the mechanical properties of GFRP should be taken into consideration.

The existing research results of GFRP mechanical properties in multi-axial stress state are mainly concerning the axial modulus, the strength, and the failure mode, but there are few research results concerning the other aspects of mechanical properties (such as transverse modulus, Poisson ratio). Comparatively, the research results of the affection of multiaxial stress state on the mechanical properties of resin are more comprehensive [23–25]. For instance, Brich has derived the functional relations between the mechanical parameters of resin and the multiaxial stress [26]. Meanwhile, mechanical properties of GFRP can be calculated by semiempirical composition formulas from the mechanical properties of resin and fiber [27].

To attain the mechanical parameters of GFRP in certain stress state, the mechanical parameters of resin in this state will be calculated primarily from its initial mechanical parameters, and then the mechanical parameters of the GFRP in the same stress state will be calculated with the composition formulas from the mechanical parameters of the fiber and that of the resin, which is calculated above.

As illuminated above, the mechanical parameters of glass fiber can be considered to be insensitive to the stress state.

The mechanical parameters of resin vary with stress state. The mechanical parameters of resin in certain stress state can be calculated by the formulas suggested by Birch:

(24) $$\begin{array}{c} E_{m}\left(P\right)=E_{m}+P\left[2\left(5-4v_{m}\right)\left(1-v_{m}\right)\right],\\ G_{m}\left(P\right)=G_{m}+P\frac{3\left(3-4v_{m}\right)}{\left(1+v_{m}\right)},\\ v_{m}\left(P\right)=v_{m}+\frac{P}{E_{m}}\frac{\left(1+v_{m}\right)\left(-1+2v_{m}\right)\left(-1+4v_{m}\right)}{v_{m}}, \end{array}$$

where −*P* = (*σ*_11_ + *σ*_22_ + *σ*_33_)/3 and −*P* is the hydrostatic pressure; *E*_*m*_, *G*_*m*_, and *v*_*m*_ are, respectively, the initial value of the elastic modulus, the shear modulus, and the Poisson ratio of the resin.

The mechanical parameters of GFRP can be calculated by the semi-empirical composition formulas as follows.

The axial modulus *E*_1_ and the transverse modulus *E*_2_:

(25) $$\begin{array}{c} E_{1}=E_{f1}V_{f}+E_{m}V_{m},\\ E_{2}=\frac{E_{f2}E_{m}\left(V_{f}+\eta_{2}V_{m}\right)}{V_{f}E_{m}+\eta_{2}V_{m}E_{f2}}, \end{array}$$

where $\eta_{2}=\left(0.2/\left(1-v_{m}\right)\right)\left(1.1-\sqrt{E_{m}/E_{f2}}+3.5E_{m}/E_{f2}\right)\left(1+0.22V_{f}\right)$ .

The shear moduli *G*_12_, *G*_23_:

(26) $$\begin{array}{c} G_{12}=\frac{G_{f12}G_{m}\left(V_{f}+\eta_{12}V_{m}\right)}{V_{f}G_{m}+\eta_{12}V_{m}G_{f12}},\\ G_{23}=\frac{G_{f23}G_{m}\left(V_{f}+\eta_{23}V_{m}\right)}{V_{f}G_{m}+\eta_{23}V_{m}G_{f23}}, \end{array}$$

where $\eta_{12}=0.28+\sqrt{E_{m}/E_{f2}}$ and $\eta_{23}=0.388-0.665\sqrt{E_{m}/E_{f2}}+2.56\left(E_{m}/E_{f2}\right)$ .

The Poisson ratios *v*_21_, *v*_12_, and  *v*_23_:

(27) $$\begin{array}{c} v_{21}=v_{f21}V_{f}+v_{m}V_{m},\\ v_{12}=\frac{v_{21}E_{2}}{E_{1}},\\ v_{23}=k\left(v_{f23}V_{f}+v_{m}V_{m}\right), \end{array}$$

where *k* = 1.095 + (0.8 − *V*_*f*_)[0.27 + 0.23(1 − (*E*_*f*2_/*E*_*f*1_))].

### 3.3. Iterative Process

**Figure 4:**
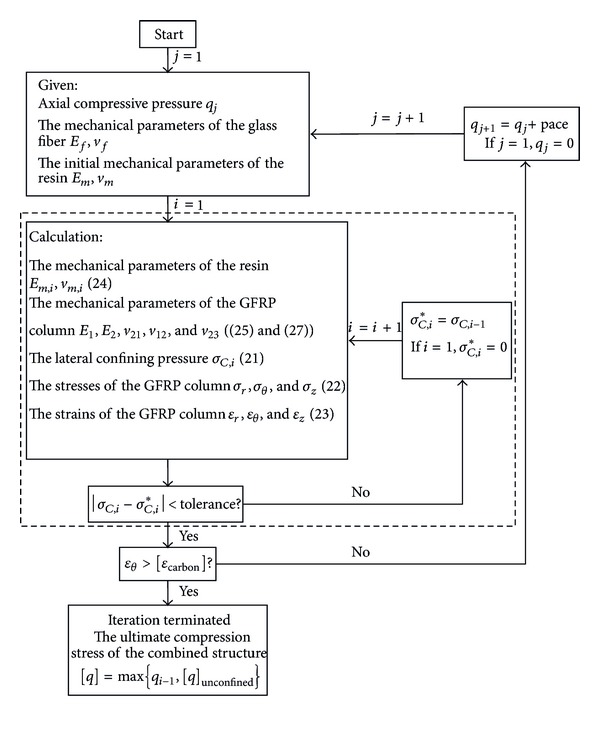
Iterative calculation process for the ultimate compressive stress of the CFRP sheet confined GFRP short pipe.

The iterative process consists of two steps. First, the stress and strain in the combined structure under certain axial compressive load are calculated by iterative method. Second, the previous step is called to calculate the ultimate compressive load of the combined structure, namely, the critical load, at which the stress and strain condition satisfies the failure criterion. The iterative process is illuminated with Figure 4, in which [*ε*_carbon_] is the ultimate strain of the CFRP sheet, while [*q*]_unconfined_ and [*q*] are, respectively, the ultimate compressive stress of the unconfined GFRP pipe and the CFRP sheet confined GFRP pipe. The iterative process is realized by programming on the computer.

## 4. Verification

### 4.1. Stress-Strain Relationship

**Figure 5:**
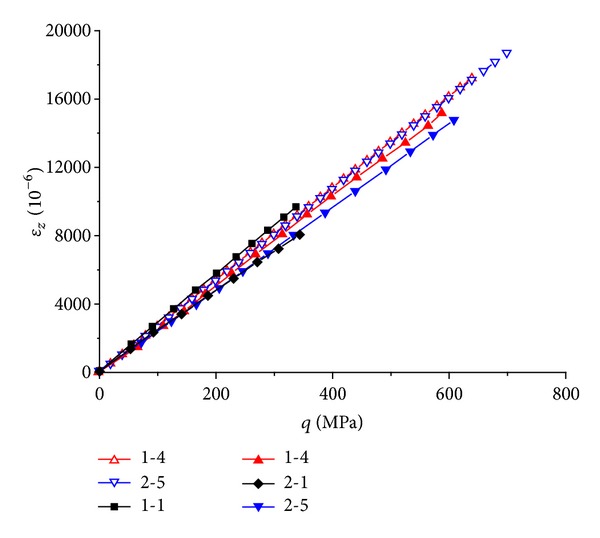
Comparison between the theoretical and experimental results on the *ε*_*z*_-*q* relation of different specimens.

The theoretical predicted *ε*_*z*_-*q* curve of each specimen is compared with its experimental result in Figure 5, where *ε*_*z*_ is the axial strain. Since the curves of the different specimens are close to each other, only the results of  1-1, 1-4,  2-1, and  2-5  are showed for graph clearness, where for 1-1  and  2-1 there are only experimental results and for the other two there are both theoretical and experimental results. The predicted responses agree well with the experimental results. There is quite small difference among the curves of the specimens. This illuminates that the presence of CFRP sheet has little effect on the axial modulus *E*_1_ of the GFRP pipe.

**Figure 6:**
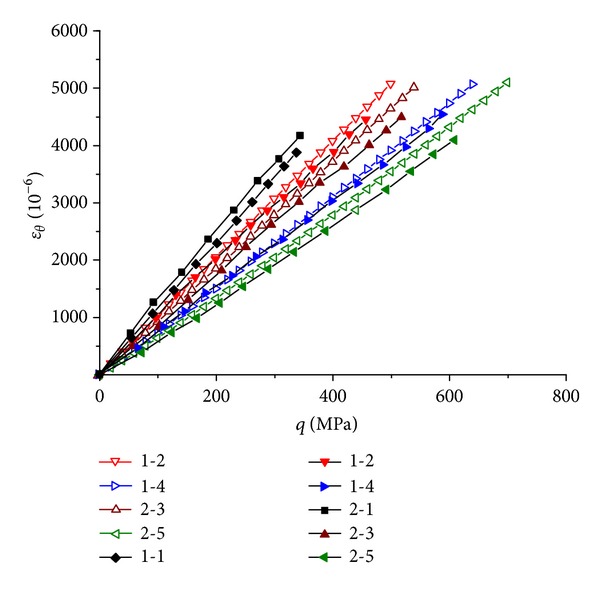
Comparison between the theoretical and experimental results on the *ε*_*θ*_-*q* relation of different specimens.

The theoretical predicted *ε*_*θ*_-*q* curve of each specimen is compared with its experimental result in Figure 6, where *ε*_*θ*_ is the circumferential strain. Only the results of 1-1, 1-2,  1-4,  2-1,  2-3, and 2-5 are showed for graph clearness, where for 1-1 and 2-1 there are only experimental results and for the others there are both theoretical and experimental results. The predicted responses agree well with the experimental results. As indicated in the previous paper, the lateral strain increase rate of the GFRP decreases gradually when the axial pressure increases; thus the *ε*_*θ*_-*q* curves should be flexural convex curves. Yet because the curvatures of the curves are quite small, this is not obvious in Figure 6. Comparing the curves of the different specimens with the same wall thickness values it can be seen that the curve slopes of the specimens with more CFRP sheet are obviously smaller than those of the specimens with less CFRP sheet. This illuminates that the presence of the CFRP sheet has relatively strong effect on the lateral modulus *E*_2_ of the GFRP pipe and restrains the lateral expansion of the GFRP pipe effectively.

These two opinions above are consistent with the research of the CFRP sheet confined GFRP short pole in the previous paper.

### 4.2. Ultimate Compressive Stress

One of the key factors affecting the predicted value of the combined structure ultimate compressive stress is the selected value for the CFRP rupture strain. For the CFRP sheet confined member, because of the factors such as local stress and brittleness of the CFRP sheet induced by the solidification, the actual rupture strain of CFRP sheet is often less than the measured value attained by tensile test of carbon fiber. 0.0051 is still adopted as the value of the CFRP sheet rupture strain [*ε*_carbon_] in this paper in consistent with the previous paper.

**Figure 7:**
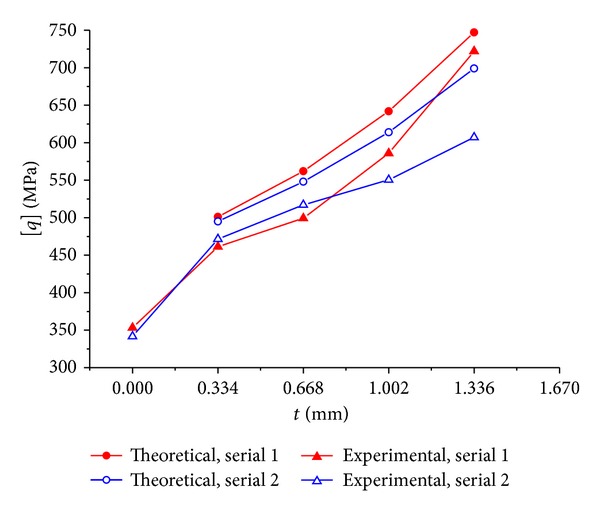
Experimental and theoretical results of the ultimate axial compressive stress of the specimens.

The predicted ultimate axial compressive stress of the specimens is showed in Table 3 and Figure 7, along with the experiment result. It can be learned from Table 3 that the average deviation of the theoretical results versus the experimental results is 9.0%, and the maximum and minimum values are, respectively, 15.1% and 3.5%. The average value and the single values of the deviation are all positive, including the fact that the theoretical results are slightly larger than the experimental results. This may be because the resin layer between the CFRP sheet and the GFRP pipe weakened the confinement and enhancement of the former to the later. If we can eliminate the effect of this factor and consider the dispersion of the experimental results, it can be realized that the theoretical values agree well with the experimental values, while this denotes that the CFRP sheet has to be wrapped tightly to the core pipe to ensure the enhancement effect.

The horizontal axis in Figure 7 denotes the total nominal thickness of the surrounding CFRP sheet, while the vertical thickness denotes the ultimate axial compressive stress of the specimen. It is seen from both the theoretical and the experimental results that the confinement of CFRP sheet can effectively heighten the ultimate axial compressive stress of the GFRP pipe, similarly with the GFRP solid section pole.

## 5. Parametric Study

When the combined structure is loaded axially, it is in the plain strain state, so its stress and strain distribution cannot be affected by the pipe height, and the geometrical parameters which affect the stress and strain distribution and its ultimate axial compressive stress are merely the outer radius *R*_*e*_, the inner radius *R*_*i*_ of the pipe, and the total thickness *t* of the CFRP sheet layers. In order to denote the relation between *R*_*e*_ and *R*_*i*_, define *β* = *R*_*i*_/*R*_*e*_. Study the influence of the geometrical parameters in the ultimate stress on the following three aspects.

### 5.1. Revarying Proportionally with *t*

**Figure 8:**
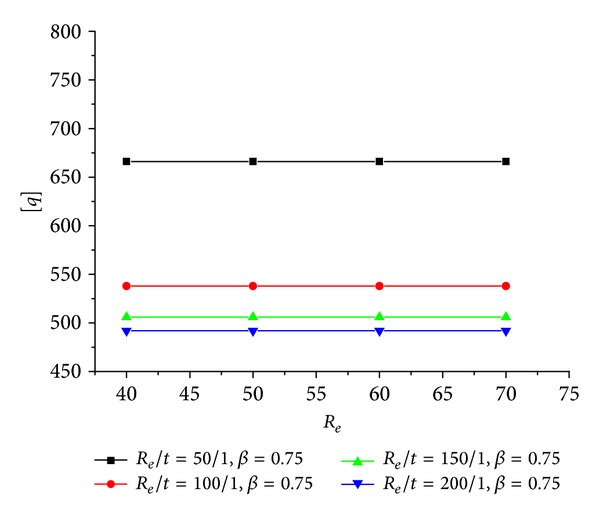
[*q*]-*R*_*e*_ relation of the CFRP sheet confined GFRP pipe when *R*_*e*_/*t* are constants.

The predicted [*q*]-*R*_*e*_ curves when *R*_*e*_/*t* are different constants are showed in Figure 8. It can be observed that when the material properties are given and normalized *R*_*e*_/*t* is constant, the ultimate axial compressive stress [*q*] keeps being a constant. Variation of *R*_*e*_ or *t* causes the variation of [*q*] by causing the variation of *R*_*e*_/*t* firstly. In addition, [*q*] is a decreasing function of *R*_*e*_/*t*.

### 5.2. Revarying Disproportionally with *t*

**Figure 9:**
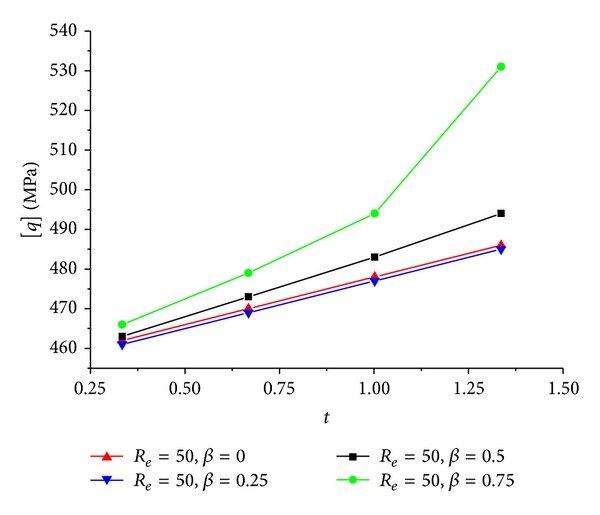
[*q*]-*t* relation of the CFRP sheet confined GFRP pipe when *β* are constants and *R*_*e*_ is 50 mm.

**Figure 10:**
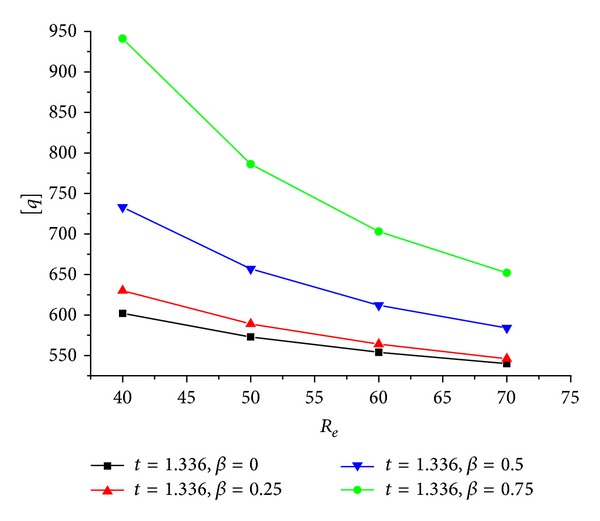
[*q*]-*R*_*e*_ relation of the CFRP sheet confined GFRP pipe when *β* are constants and *t* is 1.336 mm.

The predicted [*q*]-*t* relation when *R*_*e*_ is kept 50 mm is showed in Figure 9, and the predicted [*q*]-*R*_*e*_  relation when *t* is kept 1.336 mm (which is equal to the thickness of 8 layers of CFRP sheet) is showed in Figure 10. When *R*_*e*_ or *t* varies separately, [*q*] increases with the increase of *t* or the decrease of *R*_*e*_ and decreases with the decrease of *t* or the increase of *R*_*e*_.

**Figure 11:**
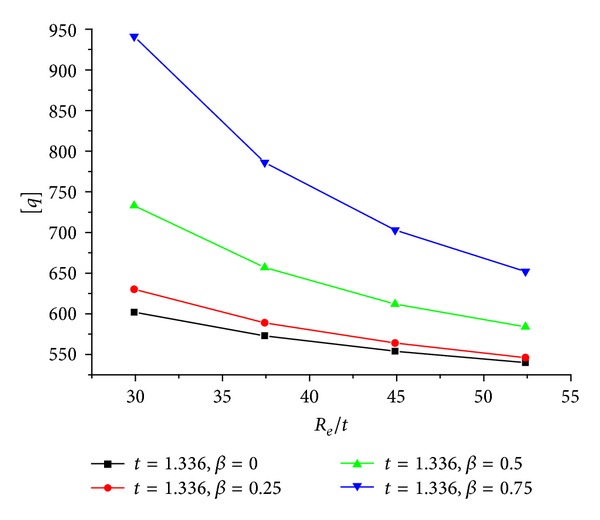
[*q*]-*R*_*e*_/*t* relation of the CFRP sheet confined GFRP pipe when *β* are constants and *t* is 1.336 mm.

The predicted [*q*]-*R*_*e*_/*t*  relation when *t* is kept 1.336 mm is showed in Figure 11. When *R*_*e*_/*t* increases, [*q*] decreases nonlinearly, and the curve tends to become horizontal. This indicates that the decrease tendency is weakening. The larger *R*_*e*_/*t*  is, the lower the sensitivity of [*q*] to the variation of *R*_*e*_/*t* is. It is easy to be inferred that, when *R*_*e*_/*t*  tends to infinity, [*q*] will tend to the axial compressive strength of the unconfined GFRP short pipe. Nevertheless, it is incorrect to infer that [*q*] tends to infinity when the unlimited increase of *t*  makes *R*_*e*_/*t*  tend to 0. This is because, in this case, the stress in the GFRP pipe is quite high, the mechanical parameters of the resin and GFRP no longer vary, and (24) is no longer fitted; consequently the calculation approach above becomes invalid.

### 5.3. Effect of *β*

**Figure 12:**
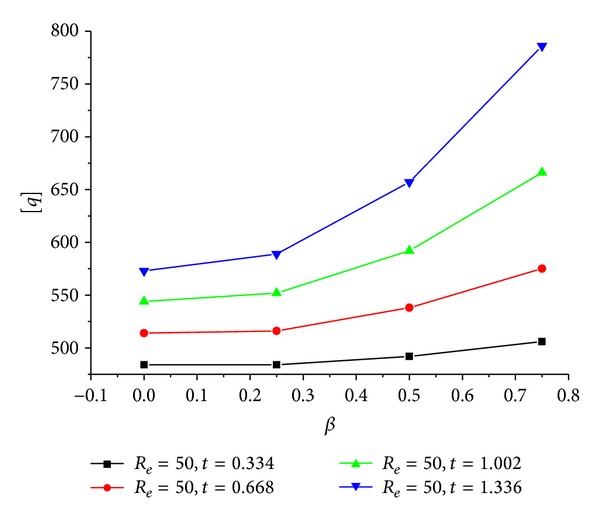
[*q*]-*β* relation of the CFRP sheet confined GFRP pipe when *t* are constants and *R*_*e*_ is 50 mm.

As showed in Figure 12, the effect of *β* on the ultimate compressive stress of the CFRP sheet confined GFRP pipe is studied taking the situation that *R*_*e*_ = 50 mm as example. It can be seen that  [*q*] increases according to *β* when *t*  is fixed. This is because the bigger *β*  is, the bigger the relative thickness of the CFRP sheet is and the stronger its confinement and enhancement to the GFRP pipe are. We can define  *E*_cf_ *t*/*R*_*e*_(1 − *β*) as the relative confinement stiffness to denote the confinement magnitude of the CFRP sheet to the GFRP pipe.

## 6. Conclusions

Similarly with the behavior of the CFRP confined GFRP solid section pole, the confinement of CFRP sheet heightens the ultimate axial compressive stress of the GFRP pipe effectively. With the increasing of the total thickness of the confining CFRP sheet, the ultimate compressive stress of the combined structure presents a nonlinear increasing trend. When the circumferential strain of the CFRP sheet achieves its rupture strain, the CFRP sheet will rupture and the GFRP pipe will transversely split consequently. In this condition, the rupture of the CFRP sheet can be seen as the sign of the failure of the combined structure.

The lateral mechanical properties of GFRP are sensitive to the stress state variation. So when the CFRP sheet confined GFRP short pipe is axially compressed the mechanical properties of the core GFRP will vary with the variation of the axial pressure and corresponding lateral pressure and cause nonlinear response of the CFRP confined GFRP short pipe. Based on the theoretical iterative calculation approach suggested in the previous paper for the design and calculation of the CFRP sheet confined GFRP short pole, the theoretical iterative calculation approach for predicting the ultimate axial compressive stress of the CFRP sheet confined GFRP short pipe is established and then realized as computer program. The transverse isotropy and the nonlinearity in multiaxial stress state of the GFRP mechanical property are considered in this approach. This approach can be used to trace the variation of the stress and strain field and material mechanical property in CFRP sheet confined GFRP pipe during the loading process and finally attain the ultimate axial compressive stress of the combined structure. The theoretical results agree with the experimental results. Additionally, the CFRP sheet confined GFRP pole in the previous paper can be seen as a special case of the CFRP sheet confined GFRP short pipe.

The accuracy of the suggested calculation approach depends mostly on the adopted values of the GFRP mechanical parameters. The adopted mechanical parameters of the GFRP are calculated with the resin mechanical parameter formulas suggested by Birch and the FRP mechanical parameter composition formulas. The two groups of formulas are applicable to the FRP which is produced by good manufacturing process and of perfect mechanical properties. For FRP of poor manufacturing quality and poor mechanical properties, in order to ensure the accuracy of the calculation, it is necessary to attain its constitutive relations under different lateral pressures by experiment.

The enhancement effect of the CFRP sheet to the GFRP pipe relates to the geometrical parameters of the combined structure.

What should be pointed out is that, as found in experiment, sometimes, if the wall of the GFRP pipe is too thin, the pipe will fail because of circumferential compression, with longitudinal splits, while the CFRP sheet has not ruptured. In this case the ultimate longitudinal compression stress of the CFRP pipe is lower than the unconfined CFRP pipe. Resultantly, the rupture of the CFRP sheet should not be seen as the failure sign of the combined structure in this situation, and the calculation approach above is not fitting. The respective condition for the two failure modes, that is, the rupture of the CFRP sheet or the circumferential compressive splitting of the GFRP pipe wall, should be ascertained by further research.

## Figures and Tables

**Table 1:** Data of the raw materials.

Data	Elastic modulus (GPa)	Poisson ratio	Volume fraction (%)	Layer thickness (mm)
Epoxy resin and curing agent	3.3	0.369	0.6	—
E-glass fiber	85.0	0.5	0.4	—
High modulus carbon fiber	200	—	—	0.167

**Table 2:** Ultimate axial compressive stress of the specimens attained by experiment.

Specimen number	Layers of the CFRP sheet	Wall thickness of the GFRP pipe (mm)	Average experimental value of the ultimate axial compressive stress (MPa)
1-1	0	4	353.3
1-2	2	4	461.4
1-3	4	4	499.2
1-4	6	4	585.9
1-5	8	4	722.1
2-1	0	8	341.7
2-2	2	8	471.6
2-3	4	8	517.1
2-4	6	8	550.6
2-5	8	8	607.2

**Table 3:** Comparison between the experimental and the theoretical results of the ultimate axial compressive stress.

Specimen number	Average experimental value of the ultimate axial compressive stress (MPa)	Theoretical value of the ultimate axial compressive stress (MPa)	Deviation of theoretical value versus experimental value (%)
1-1	353.3	—	—
1-2	461.4	501	+8.6
1-3	499.2	562	+12.6
1-4	585.9	642	+9.6
1-5	722.1	747	+3.5
2-1	341.7	—	—
2-2	471.6	495	+5.0
2-3	517.1	548	+6.0
2-4	550.6	614	+11.5
2-5	607.2	699	+15.1
